# Relationship between hemoglobinopathies and male infertility: a scoping review

**DOI:** 10.1007/s12185-024-03844-7

**Published:** 2024-09-27

**Authors:** Abdullah M. Al-Jubouri, Ahmed Eliwa, Yunes Haithm, Noof Al-Qahtani, Lolwa Jolo, Mohamed Yassin

**Affiliations:** 1 College of Medicine, QU Health, Qatar University, 2713 Doha, Qatar; 2 Department of Hematology, Hamad Medical Center, Doha, Qatar

**Keywords:** Anemia, Sickle cell, Thalassemia, Blood cells, Semen analysis, Sperm

## Abstract

Infertility is a common issue that threatens couples worldwide. Infertility can result from the male or female partner alone, or both partners. It can be due to multiple factors related to the patient’s overall health or lifestyle. Causes related to patient health can be systemic or related to gonadal dysfunction. One of the systematic causes can be hematological. The two most common hemoglobinopathies that are thought to cause infertility, especially male infertility, are sickle cell disease (SCD) and thalassemia major (TM). These two hemoglobinopathies cause male infertility through pathophysiological alterations. Specifically, they alter the oxygen carrying ability of red blood cells (RBCs), causing tissue hypoxia that affects the normal physiological process of spermatogenesis, eventually inducing infertility. Semen analyses and other systemic blood testing can be used to investigate male infertility. Both hemoglobinopathies can be helped by blood transfusions, which can then alleviate male infertility. This paper aims to explore the relationship between hemoglobinopathies (SCD and TM) and their role in contributing to male infertility, in addition to the role of blood transfusions in addressing male infertility by correcting the root cause.

## Introduction

According to the World Health Organization (WHO), infertility is defined as the inability to achieve a successful pregnancy after at least 12 months of regular unprotected sexual intercourse [1]. Infertility extends beyond being only a medical issue; but instead, it is a multidisciplinary condition that carries emotional and other social perspectives [2]. Infertility has historically been a stigmatized issue, with blame usually being placed on the woman; however, recent studies have started to explore the causes of infertility related to the male partner [3]. Infertility could further be classified into being unable to achieve successful pregnancy at all, or the cessation of a successful pregnancy after a previous successful one; primary and secondary respectively [4]. When it comes to infertility, it is thought that infertility affects about 10–15% couples worldwide, with about 50–80 million cases worldwide [5]. About 50% of the causes are attributable to female partners, 20–30% are due to male partners and a 20–30% share is due to causes related to both genders [5]. Male infertility can be a result of a multitude of factors including genetic, congenital, acquired and some remain idiopathic [6]. The aforementioned factors can affect the quality of spermatogenesis, which is crucial to maintain a man’s fertility [7]. Examples of where spermatogenesis can be hindered include faulty spermatogenesis as a result of pituitary disorders, varicoceles, or even the process of sperm transport as a result of some congenital abnormalities in the male reproductive tract [7]. It is crucial to state that for a man to be diagnosed with infertility, multiple diagnostic tests should take place looking at different parameters of the general health of the patient, such as a semen analysis and general hematological blood tests [8]. The semen analysis is substantial to look on the health of the semen itself, in terms of the number, shape and motility of the sperms themselves and how viable is the patient’s semen to help his female partner conceive [9]. Additionally, it is crucial to conduct blood serum analyses when investigating male infertility to avoid overlooking hemoglobinopathies, such as Sickle Cell Disease (SCD) or Thalassemia Major (TM) [10]. For instance, according to one study, about 72–100% of patients with SCD have at least one abnormality in a semen analysis parameter and a moderate to severe hypogonadism [11]. Moreover, according to a study published in 2017, there is a rate of hypogonadism between 40–80% in patients with TM, which can contribute to or exacerbate infertility in men [12]. This article will focus on the effect of hemoglobinopathies and their contribution to male infertility and how will parameters of sexual function change before and after undergoing blood transfusions.

## Methods

### Search strategy

The literature search was done on the 12th of July 2023 in the Google Scholar database using free text terms to make sure there was a broad search strategy. Terms included within the search: ‘Male infertility’, ‘Hemoglobinopathies’, ‘Sickle Cell Disease’, ‘Thalassemia major’, ‘Semen Analysis’, and ‘Blood transfusion’. Free text terms utilized: The search strategy was limited to human studies. The search was not restricted by language or time frame, as long as the paper was complete and not a solely abstract article. All of the resulting studies were then transferred to EndNote X9, where duplicates were identified and removed.

### Eligibility criteria (inclusion and exclusion)

Studies related to the main keywords mentioned above were included provided they had information about the pathophysiology or treatment options for the diseases. Research articles were excluded from our review if they were (1) incomplete or abstract only; (2) were published in a different language; (3) the same sample was a duplicate or similar between studies. If more than one study used the same sample, only the article with the older publish date was included.

### Study selection and screening

Titles and abstracts were reviewed. The whole text of the selected papers was then inspected and the key points were obtained from each paper and arranged into several points to be written about in this scoping review.

### Outcomes

Our primary outcomes in this scoping review is to outline the pathophysiological principles that cause the sequelae of infertility in the male partners who are affected by hemoglobinopathies. In this study, we focused only on two hemoglobinopathies, which were Sickle Cell Disease (SCD) and Thalassemia Major (TM). We also looked on the changes within the semen analysis parameters before and after receiving blood transfusions and how could it be promising for future research. We utilized data from 2 published papers which compared the differences in semen analysis before and after blood transfusion. There were a total of 18 patients with SCD and 10 patients with TM which were included.

## Results

It is crucial to identify how patients who are affected with SCD and TM will benefit after being transfused with blood and how the semen analysis parameters improved pre has and post blood transfusions. It is important to recall that male patients with SCD and TM are affected by having reduced sperm quality parameters within the semen analysis, in addition to decreased serum levels of reproductive hormones [13–15]. According to one study, patients who were affected with SCD, have showed a clear improvement within 7 days after packed red cell transfusion [16]. It compared two types of RBC transfusion, either as SCD exchange (where patient’s blood was exchanged with new blood), or SCD top up (whereby two packed RBC units were added with IV access to the patient’s blood) and SCD total was an average of both transfusion methods. The data were then compared within the table with all measured parameters and 1 resembling “before transfusion”, whereas 2 resembled “after transfusion”.The transfusion has improved the hematological analysis, reflected by the positive change in the levels of hemoglobin [16]. In fact, the levels of serum Hb went from about 8.5 g/dL to 10.5g/dL [16]. Moreover, regarding the improvement that was seen within the semen analysis parameters, it showed an improvement in sperm motility, morphology and sperm count [16]. The improvement was evident upon the sharp increase within sperm count from 87.4 million/ml to 146 million/ml and motility jumped from 29.3% to 67.4% [16]. Additionally, there was an improvement in the serum levels of testosterone, FSH and LH [16]. When it comes to numbers, Testosterone levels changed from 12.3 nmol/L to 14.2nmol/L, LH from 4.4 U/L to 5.7 U/L, and FSH from 5.4 U/L to 6.6 U/L [16]. These hormones are crucial in all stages of spermatogenesis as they help the existent sperms become healthier and improve their quality. Additionally, these hormones are required to fasten the terminal stages of spermatogenesis [17]. All these positive changes to the spermatozoa could be explained by the fact that blood transfusion would help improve tissue oxygenation by increasing blood flow in the reproductive tract microcirculation [16]. Adequate and effective blood transfusion would also help control optimal levels of iron and reduce iron toxicity, slowing down the process of ineffective erythropoiesis [16, 18]. It is essential to recall that iron is essential to help support sperm cells within the semen to keep it preserved even after ejaculation [18]. Further information can be obtained from Table 1 [16].

**Table 1:** Hormonal and sperm parameters in patients with sickle cell disease before and after transfusion

Parameter	SCD-exchange *n* = 8	SCD-top-up *n* = 10	SCD-total *n* = 18
Age (year)	20.33 ± 2.16	21 ± 3.67	20.72 ± 2.88
LH-1 U/L	3.83 ± 0.9	4.8 ± 0.97	4.4 ± 1.09
LH-2 U/L	5.0 ± 0.55*	5.9 ± 0.85*	5.74 ± 0.76*
FSH-1 U/L	5.5 ± 1.05	5.57 ± 1.49	5.56 ± 1.28
FSH-2 U/L	7.21 ± 0.93*	7.6 ± 1.29*	7.43 ± 1.12*
Testost-1 (nmol/L)	12.1 ± 1.45	12.48 ± 1.42	12.33 ± 1.41
Testost-2 (nmol/L)	14.26 ± 1.32*	14.28 ± 1.22*	14.27 ± 1.24*
Hb-1 g/dL	7.9 ± 1.15	8.9 ± 1.19	8.4 ± 1.22
Hb-2 g/dL	10.5 ± 0.4*	10.8 ± 0.5*	10.7 ± 0.45*
Ferritin	439 ± 557	847 ± 705	668 ± 597
Sperm count-1 (M/ml)	85.33 ± 15.10	90.56 ± 22.52	87.44 ± 18.22
Sperm count-2 (M/ml)	24.82 ± 11.25*	47.42 ± 24.56*	42.87 ± 20.45*
Volume-1 (ml)	3.20 ± 0.32	3.25 ± 0.44	3.23 ± 0.38
Volume-2 (ml)	3.45 ± 0.37**	3.32 ± 0.51	3.36 ± 0.45
Total PM (M/ml)-1	41.80 ± 7.5	43.27 ± 7.7	42.8 ± 7.5
Total PM (M/ml)-2	12.92 ± 3.7*	17.53 ± 9.16*	14.81 ± 7.2*
RPM (M/ml)-1	27.48 ± 7.7	29.26 ± 8.75	28.55 ± 8.3
RPM (M/ml)-2	53.56 ± 18.5*	67.42 ± 29.1*	60.88 ± 25.2*
NPM (M/ml)-1	25.38 ± 10.8	21.62 ± 10.25	23.2 ± 10.52
NPM (M/ml)-2	27.86 ± 12*	29.74 ± 9.15*	28.58 ± 10.7*
Immotile-1 (M/ml)	22.88 ± 7.6	23.44 ± 9.2	23.20 ± 8.5
Immotile-2 (M/ml)	40.45 ± 17.26*	30.88 ± 13.45*	35.60 ± 15.78*
Normal morphology-1%	32.7 ± 5.43	31.44 ± 5.43	31.95 ± 5.27
Normal morphology-2%	57.2 ± 3.3*	51.56 ± 1.13*	53.88 ± 3.57*

*1* Before, *2* After PCTx, *SCD* Sickle cell disease, *LH* Luteinizing hormone, *FSH* Follicle-stimulating hormone, *Hb* Hemoglobin, *RPM* Rapid progressive sperm motility, *NPM* Normal progressive motility

^*^P < 0.05 exchange transfusion group (ETx) versus top-up group (TTx), *P < 0.05 after versus before PCTx

The results are almost similar when it comes to patients with TM. According to one study that looked on the effects of transfusion on different parameters of the semen analysis, it has shown that the effects are almost identical, showing the same levels of improvement in semen analyses and the elevation of crucial reproductive hormones within the serum [19]. Sperm counts have increased from 57.8 million/mL to 166 million/mL and motility went from 20.6% to 79.7% [19]. Additionally, Hb levels also increased from 8.7 g/dL to 11.1 g/dL and Testosterone increased from 16.5 nmol/L to 20 nmol/L [19]. It is crucial to mention that all these changes are also reflected after the enhanced level of oxygenation, which resulted in improved secretion of pivotal hormones that help orchestrate the process of spermatogenesis [19]. More details can be seen in Table 2 [19].

**Table 2:** Hormonal and sperm data before versus after blood transfusion in thalassemic men (*n* = 10)

Parameter	Before	After	*P* value
Hemoglobin (g/dL)	8.7 ± 0.86	11.1 ± 0.82	0.000046
LH (IU/L)	2.5 ± 1.65	3.8 ± 1.87	0.049
FSH (IU/L)	3.3 ± 2.45	4.4 ± 2.27	0.008
T (μmol/L)	16.5 ± 8	20 ± 8.8	0.0018
Basal GH (ng/mL)	0.88 ± 1.3	ND	
Peak GH (ng/mL) after clonidine	6.7 ± 2.66	ND	
IGF-1 (ug/L)	173 ± 46	214 ± 61	0.003
Seminal parameters			
Volume (mL)	1.65 ± 0.78	2.1 ± 0.58	0.0406
Total sperm count (million/mL)	57.838.3	166 ± 132	0.0059
Rapid progressive motility (%)	23.9 ± 15.3	64.9 ± 12.6	0.0026
Normal forms (%)	41.7 ± 17.6	48.3 ± 12.4	0.045

It is crucial to mention that in both previous data sets (for SCD and TM), the researchers have not discussed whether or not the effects of the transfusion have lasted transiently or the semen analysis parameters have regressed back to pre-transfusion numbers. This principle should be thought of when reading the results of blood transfusion for patients of both SCD and TM.

## Discussion

It is essential to understand the consensus in which solely these two hemoglobinopathies were the main focus of this paper. This can be explained by the fact that although other forms of hemoglobinopathies can exist, the two hemoglobinopathies selected in this paper (SCD and TM) remain the most common forms of hemoglobinopathies in terms of prevalence and clinical significance [20]. It is crucial to understand the pathogenesis for each. Both SCD and TM occur due to anomalies within the Beta globin gene on chromosome 11 (11p 15.15) [21]. Whilst TM occurs due to an array of mutations that result in a quantitative deficiency of structurally viable beta globin genes, SCD happens due to a point mutation that substitutes an amino acid within the 6th position [21]. SCD is an autosomal recessive disorder that cause abnormalities in the Beta globin gene [22]. Upon deoxygenation of the hemoglobin S (sickle hemoglobin), a replacement in the 6th position of the beta globin gene takes place which incurs SCD; the latter is the replacement of glutamic acid with valine [23]. This leads to the aggregation of these hydrophobic molecules interacting with each other, consequently resulting into polymerization into larger molecules which can occlude blood vessels [23]. Not only does the polymerization process changes the shape of the Red Blood Cells (RBCs), it also exerts some mechanical force, affecting the RBCs’ membrane integrity and resulting in the loss of RBC membrane material [24]. This can be measured in serum by quantifying the levels of RBC derived micro particles [24].

All these aforementioned pathophysiological changes lead to the deterioration of the RBCs lifespan from what is expected to be 120 days in normal people, to only about 5–15 days in people affected with SCD [25]. This leads to a reduced oxygen carrying capacity of the RBCs and results in severe anemia in a big number of people affected with SCD [25]. When talking about the accelerated hemolysis and abnormal RBC’s morphology in SCD patients, one of the possible clinical manifestations can be priapism [26]. Priapism is a prolonged erection of the penis which is usually unrelated to sexual activity and is typically painful [27]. Due to sickling and hemolysis of RBCs in patients with SCD, sickled cells will usually cause vaso-occlusion of small arteries of the penile erectile tissue (corpora cavernosa) which hinders normal blood flow [28, 29]. This could consequently lead to tissue hypoxia and thus irreversible penile fibrosis and resultant erectile dysfunction if not treated promptly [30].

When it comes to TM, it is also a hereditary disorder that incurs due to erroneous synthesis of globin protein [31]. However, the exact pathogenesis follows a slightly different route to that of SCD. First, it is essential to state that TM is described in literature by its inability to synthesize RBCs and resulting in long term anemia [32]. In fact, TM is the most common autosomal recessive hemolytic anemia that takes place due to ineffective synthesis of hemoglobin [33]. It is pivotal to recall that hemoglobin synthesis depends on the presence of two alpha and two beta globin chains, which combine together along with a molecule of heme containing an iron Fe^2+^ ion to form a hemoglobin molecule [34]. TM is characterized by a defect in the beta chain of the globin molecule, resulting in faulty hemoglobin [35]. Generally, Thalassemia can present in a spectrum of severity, with TM being the most severe and is a life-long anemia that is also known as transfusion dependent thalassemia [36]. TM’s severity can allow it to exert substantial effects on the quality of life for patients affected and have its effects extend beyond only being a severe anemia, to affecting many organs and metabolic processes around the body [37]. Organ damage induced by TM is usually due to the iron overload state that incurs due to the chronic blood transfusions for TM patients [38]. Organs affected can include heart, endocrine organs and the liver, all of which are crucial in maintaining healthy sexual habits and fertility [38]. However, it is also crucial to mention that another version of thalassemia does exist that may be less severe and may not necessitate blood transfusions, also known as non transfusion dependent thalassemia which may present in a less intense clinical course [39].

Both of these hemoglobinopathies mentioned above can contribute to male infertility [40, 41]. When trying to find what is common between SCD and TM and why could both contribute to male infertility, the effect of both disorders on male infertility could easily be summarized by one single word; hypoxia [42]. Hypoxia in itself means a decreased oxygen pressure in the organ which can possibly be due to a lower oxygen exchange between the surrounding environment and the vascular bed [43]. Since it is well known that RBCs are the oxygen carrying vehicles within the body, a defected RBC can have a deteriorated oxygen carrying ability [44, 45]. It is well established that one out of the numerous physiological processes of the body in which oxygen abundance is essential is spermatogenesis; the process by which sperms are made [46, 47]. This process can be slightly compromised by reversible dysfunction should the person be exposed to some degree of hypoxia [48].

Precisely, tangible parameters that could be affected in semen analyses of males who are subjected to hypoxia can include a decreased sperm count, motility, density and survival time; providing evidence of decreased overall sperm quality upon exposure to hypoxia [49]. It is crucial to mention that the sample of patients in which this result was obtained from had the main hypoxic insult be the exposure to a high altitude over a sustained period of time; another mechanism in which hypoxia can be induced [49]. The purpose of mentioning another mechanism of hypoxia is just to show that the main insult that results in the decline of sperm function is the hypoxia itself, which could be achieved by the mechanism above. However, with regards to the focus of this paper, it is well established that the same decline in semen analyses is observed in males who are affected with SCD and TM [13]. Not only are their semen analyses’ parameters declined, but also the serum levels of reproductive hormones such as Testosterone, FSH and LH are all deteriorated in patients who have SCD and TM compared to individuals who do not [14, 15].

**Fig. 1:**
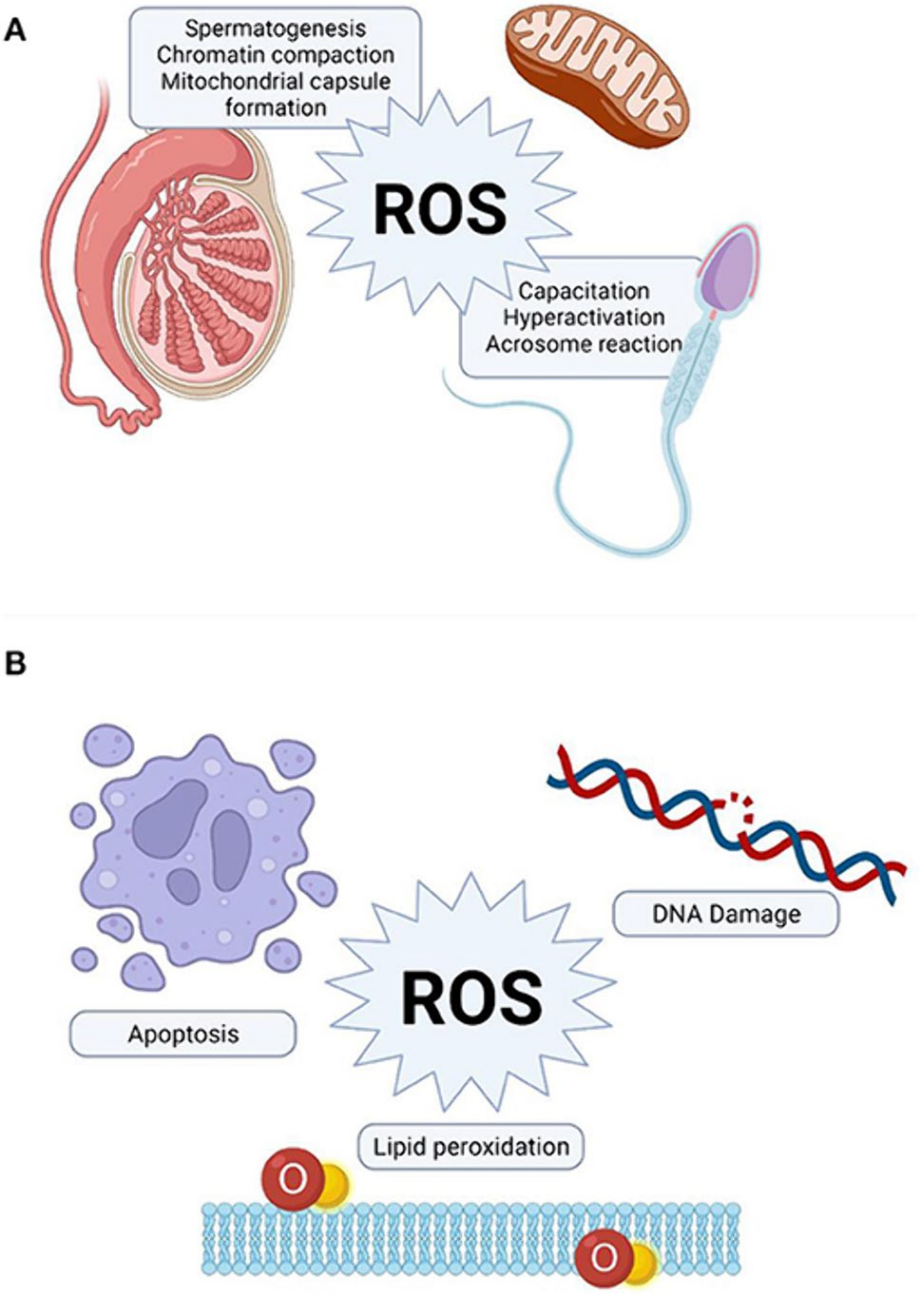
The physiological and pathological effects of ROS [56]: This figure was obtained from the article with the reference below. It is under a creative commons license. Reference DOI: https://doi.org/10.3389/frph.2022.822257

The danger of hypoxia is that it leads to the process of Oxidative Stress (OS) that incurs the production of Reactive Oxygen Species (ROS), which exerts the effects on male fertility [50]. In fact, one study stated that ROS can be found in semen samples of 25–40% of infertile men; which is alarming [51]. The sperm cells are prone to getting damaged by OS due to numerous reasons, one of which is the fact that their plasma membranes are rich of polyunsaturated fatty acids, which are susceptible to peroxidation reactions that can damage the spermatozoa’s membrane affecting its motility [52]. Not only does the OS target the sperm motility, it also targets the viability of the DNA that is housed within the sperm’s nucleus and accelerates the apoptosis of the germ cells [53]. This latter insult causes the deterioration in the sperm’s quality and quantity over time [54]. It is also crucial to mention that although some body cells are able to correct the damage that is induced by OS, spermatozoa are not able to due to them being deficient in the enzymes that are responsible for correction [55]. However, although ROS is known to impose a negative role on the cellular pathways within the body, it can also have some important physiological functions within spermatogenesis [56]. For instance, ROS can be useful in aiding formation of the mitochondrial capsule, as well as capacitation of spermatozoa [56]. Figure 1 can illustrate the physiological and pathological roles of ROS [56].

When it comes to treat these two types of hemoglobinopathies, the treatment firstly has to focus on the removal of the insulting agent that was stated and explained above; hypoxia. As mentioned above, the hypoxia is a resultant of a defective RBC that is not able to fulfill its action in carrying oxygen around the body and delivering it to spermatozoa. Regarding SCD, the mainstay of treatment is either blood transfusion or hydroxyurea therapy [57]. The transfusion of blood is essential, as it reduces the amount of sickle hemoglobin (HbS), in addition to increasing the oxygen carrying ability [58]. However, one important thing to pay attention to is the fact that the transfusion should be administered after careful calculations to give the right amount of blood and avoid excessive transfusion that could impose unwanted sickling, which could be vaso-occlusive [58]. On the other hand, when it comes to treating TM, the best treatment for TM is the Hematopoietic Stem Cell Transplantation (HSCT), which outweighs in its clinical usefulness and long-term cost effectiveness its alternatives, such as chelation therapy [59]. However, the popular and more accessible treatment method for people who suffer TM is the regular blood transfusion and chronic iron chelating medications [60]. It is important to mention that the blood transfusion should also be administered after careful hematological analysis to avoid inducing iron overload-related complications to the tissues of the body [60]. One of the complications that could incur after chronic transfusion is hypogonadism secondary to iron overload [61].

Pharmacological treatments of both hemoglobinopathies can be also useful to alleviate some of the effects of these two diseases. Firstly, Hydroxyurea is the most commonly prescribed medication for patients with SCD and to a lesser degree to TM patients [62]. It is thought that hydroxyurea’s mechanism of action is completely unknown yet [63], but it functions mainly to stimulate fetal hemoglobin which has positive effects on the hemoglobin indices within the blood, as well as an improved erythropoiesis process [64]. Although Hydroxyurea is the mainstay of pharmacological managements for these two hemoglobinopathies, other drugs can also be used to help induce the formation of fetal hemoglobin [65]. Such medications include butyrates, DNA methyltransferase inhibitors and Thalidomide derivatives [66]. Regarding studies that show efficacy of hydroxyurea, a meta-analysis have concluded that hydroxyurea have helped patients with thalassemia major achieve higher hemoglobin levels and less transfusions required [67]. Similarly, a systematic review about the effect of Hydroxyurea on SCD showed that people who took the medication had less pain crises, less hospitalizations and less need for frequent transfusions compared to patients who did not [68]. Despite the fact that Hydroxyurea helps SCD, one study have shown that Hydroxyurea therapy can cause a reversible azoospermia that is likely to correct upon cessation of therapy [69]. The same study suggested the possible use of cryopreservation of spermatozoa prior to initiation of therapy to insure that sperms remain viable [69].

Although patients with SCD and TM are surviving into four or five decades of their lives, it is important that physicians remember that many of those patients will express issues related to sexual maturation and physiological growth since their adolescence [70]. Hence, it is crucial to start developing alternative solutions for these hemoglobinopathies that can increase the quality of life for these patients. What this research have shown could be used as basis for future research to explore the effectiveness of blood transfusions as means of treating male infertility by correcting these underlying causes, instead of treating the outcome; which is infertility. Researchers should keep in mind that although blood transfusions will be beneficial for these patients, further research should be warranted to explore the duration and intervals of blood transfusion that SCD and TM patients require for relief of their symptoms. Moreover, further research is needed to explore the long term effects and solutions of complications related to blood transfusion and avoiding the detrimental effects of iron overload [71].

It is crucial not to overlook the social aspect of these two hemoglobinopathies and the possible resultant infertility on the couple and its profound effects [72]. These disorders can cause a multitude of health challenges due to the emotional and social burden it can bring [73]. The impact can be namely due to the decreased quality of life between couples secondary to decreased sperm quality and quantity leading to infertility [74]. The distress caused by the latter is likely to cause some stigma and social anxiety and causing social withdrawal [75]. Along with medical treatment for these two hematologic disorders, psychological and social therapy sessions can provide substantial help for couple affected [75].

Regarding this paper, there could be some limitations that researches of this study have identified. First, there was not a clear criteria of selection of papers within this study and papers were selected without a clear consensus as to why have they been selected. Second, there was no period for the selection of papers and the timeframe was kept broadly open. The second limitation can also lead to the third limitation that is some of the papers that were selected might have been too old for the date of writing this article, meaning that some of the information could be outdated or replaced by new data. One more limitation can be that there was a limited sample size of patients from the two papers from which the data was obtained. Although the selection of only one study representative of each of the two groups (SCD and TM) is a limitation, the two included tables from the two papers used was because we believed that the parameters were reported based on the same protocols and outcomes, ensuring more reliable results. A final limitation that could be stated is that this paper did not explore the relationship between all hemoglobinopathies and male infertility; instead, it only focused on SCD and TM.

## Conclusion

This research explored the relationship between hemoglobinopathies (specifically sickle cell disease and thalassemia major) and male infertility. Male infertility was shown to result from various factors affecting spermatogenesis, including genetic, congenital, acquired, and idiopathic causes. Diagnostic tests such as semen analysis and blood tests are essential for diagnosing male infertility, assessing sperm quality, and identifying underlying health conditions like hemoglobinopathies. Sickle cell disease and thalassemia major can contribute to male infertility by inducing hypoxia, reducing sperm quality, and decreasing reproductive hormone levels. Although hemoglobinopathies are primely due to genetic abnormalities, it is the resultant hypoxia that incurs due to defective RBCs functionality that precipitates male infertility. Hypoxia, resulting from the defective oxygen-carrying capacity of affected red blood cells, leads to oxidative stress, which damages sperm cells' viability, motility, and DNA. Treatment options for hemoglobinopathies include blood transfusions to alleviate hypoxia and improve hematological parameters. Studies have shown that transfusions positively impact semen analysis parameters, including sperm count, motility, and morphology, as well as serum reproductive hormone levels. By enhancing tissue oxygenation, blood transfusions aid in spermatogenesis and help regulate iron levels. These findings highlight the potential of blood transfusions in improving male infertility associated with hemoglobinopathies and suggest the need for further research to address complications of the transfusion-resultant iron overload. Ultimately, this research highlights the importance of considering and treating underlying causes of male infertility rather than focusing solely on the outcome.

## Data Availability

This research utilized data from published papers obtained through PubMed and Google Scholar.
